# Photo-Crosslinked Hyaluronic Acid/Carboxymethyl Cellulose Composite Hydrogel as a Dural Substitute to Prevent Post-Surgical Adhesion

**DOI:** 10.3390/ijms23116177

**Published:** 2022-05-31

**Authors:** Yin-Cheng Huang, Zhuo-Hao Liu, Chang-Yi Kuo, Jyh-Ping Chen

**Affiliations:** 1 Department of Neurosurgery, Chang Gung Memorial Hospital, Linkou, Kwei-San, Taoyuan 33305, Taiwan; ns3068@gmail.com (Y.-C.H.); b8402022@gmail.com (Z.-H.L.); 2 Department of Medicine, Chang Gung University, Kwei-San, Taoyuan 33302, Taiwan; 3 Department of Chemical and Materials Engineering, Chang Gung University, Kwei-San, Taoyuan 33302, Taiwan; onesky1997@gmail.com; 4 Craniofacial Research Center, Chang Gung Memorial Hospital, Linkou, Kwei-San, Taoyuan 33305, Taiwan; 5 Research Center for Food and Cosmetic Safety, College of Human Ecology, Chang Gung University of Science and Technology, Taoyuan 33305, Taiwan; 6 Department of Materials Engineering, Ming Chi University of Technology, Tai-Shan, New Taipei City 24301, Taiwan

**Keywords:** hydrogel, dural substitute, hyaluronic acid, carboxymethyl cellulose

## Abstract

A dural substitute is frequently used to repair dura mater during neurosurgical procedures. Although autologous or commercially available dural substitutes matched most of the requirements; difficulties during dural repair, including insufficient space for suturing, insufficient mechanical strength, easy tear and cerebrospinal fluid leakage, represent major challenges. To meet this need, a photo-crosslinked hydrogel was developed as a dural substitute/anti-adhesion barrier in this study, which can show sol-to-gel phase transition in situ upon short-time exposure to visible light. For this purpose, hyaluronic acid (HA) and carboxymethyl cellulose (CMC), materials used in abdominal surgery for anti-adhesion purposes, were reacted separately with glycidyl methacrylate to form hyaluronic acid methacrylate (HAMA) and carboxymethyl cellulose methacrylate (CMCMA). The HA/CMC (HC) hydrogels with different HA compositions could be prepared by photo-crosslinking HAMA and CMCMA with a 400 nm light source using lithium phenyl-2,4,6-trimethylbenzoylphosphinate as a photo-initiator. From studies of physico-chemical and biological properties of HC composite hydrogels, they are bio-compatible, bio-degradable and mechanically robust, to be suitable as a dural substitute. By drastically reducing attachment and penetration of adhesion-forming fibroblasts in vitro, the HC hydrogel can also act as an anti-adhesion barrier to prevent adhesion formation after dural repair. From in vivo study in rabbits, the HC hydrogel can repair dural defects as well as protect the dura from post-operative adhesion, endorsing the possible application of this hydrogel as a novel dural substitute.

## 1. Introduction

The repair of dura mater is a crucial issue during neurosurgical procedures, which commonly involves the removal or perforation of dura mater. Traditionally, primary dural repair using autologous tissues harvested from the patient’s own pericranium or fascia latae is regarded as a standard procedure [1,2]. Although these tissues are desirable with minimal inflammatory response and mimic the native dura, their use is limited by availability and harvest site morbidity. Dural defects located in regions where surgical stitches are difficult (e.g., skull base) represent additional challenges. The failure of dural healing may cause catastrophic complications such as central nervous system infection or failed wound healing [3,4].

Many materials have been considered human dural substitutes and commercially available dural substitutes are highly diversified [5]. Xenograft materials, such as bovine pericardium and pig small intestine submucosa, have been considered [6]. Although they do not require harvesting from donors and are readily available, complications such as inflammatory reactions, easy resorption and fast degradation limit their use. On the other hand, numerous natural and synthetic materials have been proposed as the base material in dural substitutes, including cellulose, collagen, silicone, polytetrafluoroethylene, poly(glycolic acid) and L-lactic acid-ε-caprolactone copolymer [7,8,9]. The dural substitute Preclude™, fabricated from a synthetic polymer expanded polytetrafluoroethylene film, is non-degradable but provides a long-term barrier to cerebrospinal fluid leakage. With its permanent presence in the body, this dural substitute often leads to fibrosis that interferes with the proximal cortex and surrounding tissues [10]. The other synthetic graft Ethisorb™ is a composite of Polyglactin 910 and polydioxanone film, which is fully resorbable following neoduralization [11]. However, poor handling, as well as a poor approximation of the mechanical properties of the dura matter, complicate the clinical use of synthetic grafts. These products are advantageous for the coverage of dural defects either with or without suturing. Nevertheless, there is still a need for an advanced design of a dural substitute, where the dural substitute can seal the dural defect in a water-tight fashion whenever possible, as well as minimize post-operative adhesion formation in the surgical site [12,13].

Dural repairs could be accomplished with hydrogels, and some off-label use of hydrogel products of biological origin (e.g., fibrin glues, gelatin, collagen, etc.) have also been proposed for dural sealing [14]. The biological adhesive DuraSeal Dural Sealant System is a commercially available polyethylene glycol (PEG) hydrogel sealant for watertight dural sealing [15]. Although this material can meet the requirement of immediate watertight closure with sutureless dural repair, it cannot be used alone as a sutureless dura sealant in clinical practice. Therefore, it is desirable to develop an easy-to-use biodegradable hydrogel sealant as a dural substitute, which can be endowed with desirable properties such as quick set time, good mechanical strength, low cytotoxicity and resistance to adhesion to the dura [16].

The gold standard for dural repair is using autologous tissue, such as pericranium, temporal muscle or fascia lata [17]. However, due to reasons such as tissue loss, prolonged surgical time or insufficient space for suturing, there is a need for non-autologous material as dural substitutes, ranging from the animal pericardium, and intestine to synthetic collagen [1]. There are pros and cons associated with these non-autologous dural substitute materials. In an animal study, the non-autologous graft was reported to be as effective as autologous grafts [17]. In general, a dural substitute should be biocompatible, watertight, porous and biodegradable. Hydrogel formed from alginate has been suggested as a substitute for dura mater, which can be applied in liquid form and molded to contours of the tissue, as well as creating a watertight seal upon gelation [18]. Alternatively, blended polymer solutions of HA and CMC were applied directly as a film over the anastomosis to prevent intestine anastomosis adhesion [19]. Hyaluronic acid (HA) is a natural polysaccharide with repetitive disaccharide units of D-glucuronic acid and N-acetyl-glucosamine. As a key component in the extracellular matrix of connective tissues with high water adsorption and retention ability, HA is highly biocompatible and does not elicit foreign body reactions [20]. In addition, exogenous HA can inhibit fetal fibroblast proliferation and play a role in reducing scar formation and fibrosis during the early stages of wound healing [21]. With this and other unique physicochemical properties, HA has become one of the most widely used biomaterials to prevent post-operative adhesion in various forms [22]. However, the main disadvantages of HA-containing anti-adhesion products are their fast degradation rates and poor mechanical properties [23]. Specifically, HA-grafted polycaprolactone nanofiber membranes can prevent peritendinous adhesion following tendon repair surgery in rabbits [24]. Thermo-responsive in-situ forming hydrogels of HA were used as barriers to prevent post-operative peritendinous adhesion [25]. Similarly, using adipic acid dihydrazide as a crosslinking agent, an in-situ forming HA hydrogel was used to prevent epidural fibrosis after laminectomy in rabbits [26]. The application of thiol-modified HA hydrogels was shown to be effective in reducing tissue adhesion after flexor tendon surgery in rabbits [27].

The cellulose derivative CMC is water-soluble and can be with excellent anti-adhesion properties by forming physical barriers at the surgical site that is prone to adhesion formation [28]. Compared with HA, CMC shows a slower absorption rate in the body, making it a preferred candidate to develop anti-adhesion biomedical devices [29]. Therefore, CMC is now widely used as a major component of commercial anti-adhesion barriers (e.g., Seprafilm^®^ and Intercoat^®^), since it can also inhibit the proliferation and migration of fibroblasts. By blending with gelatin, a CMC/gelatin composite hydrogel was developed as an anti-adhesion barrier through radiation-induced crosslinking [30]. However, it should be noted that CMC is biodurable, since mammalian enzymes cannot degrade cellulose from plant origin [31]. Therefore, by co-crosslinking CMC with HA, which is biodegradable by hyaluronidase, the HA/CMC hydrogel is expected to show enzyme-mediated degradability but still preserve the non-adhesive nature of CMC. In clinical practice, the blend of sodium hyaluronate and CMC was found to demonstrate anti-adhesion properties in various forms [32,33,34]. Therefore, HA and CMC were selected as the base polymers to make HC composite hydrogels in this study, which can be prepared from HAMA and CMCMA by reagent-free light-induced crosslinking.

The biodegradable adhesion barrier Seprafilm is a synthetic membrane composed of sodium salt of hyaluronic acid (HA) and carboxymethyl cellulose (CMC), which has been used by surgeons in abdominal and gynecological fields [35,36]. It is also used successfully in clinical craniectomy as a dural substitute, by placing a layer of Seprafilm over the dura as an onlay, in addition to acting as an anti-adhesion barrier [37]. Minimal adhesion to the underlying Seprafilm/dural layer was reported and the HA/CMC film has been incorporated into the surrounding dural plane without noticeable post-operative complications. With this in mind and inspired by the dental filling technology, photo-crosslinked hyaluronic acid (HA)/carboxymethyl cellulose (CMC) hydrogel was developed in this study as a dural substitute. The HA/CMC hydrogel is facilely prepared in situ at the surgical site after dural repair, by co-crosslinking hyaluronic acid methacrylate (HAMA) and carboxymethyl cellulose methacrylate (CMCMA) with 400 nm visible light [38]. The hydrogel is expected to act as a physical barrier to prevent post-operative adhesion and remains in place during active healing. It could be fully resorbed after healing is complete. The simple sutureless technique for application may also allow its precise placement during the cranial procedures with light-induced sol-to-gel phase transition of a HAMA/CMCMA polymer solution. This photo-crosslinked HA/CMC (HC) hydrogel was demonstrated to be biocompatible, degradable and mechanically robust, as well as with the ability to prevent fibroblast penetration and adhesion in vitro, which can prevent post-operative adhesion after brain surgery in vivo.

## 2. Results

### 2.1. Synthesis and Properties of HA/CMC Hydrogel

**Figure 1:**
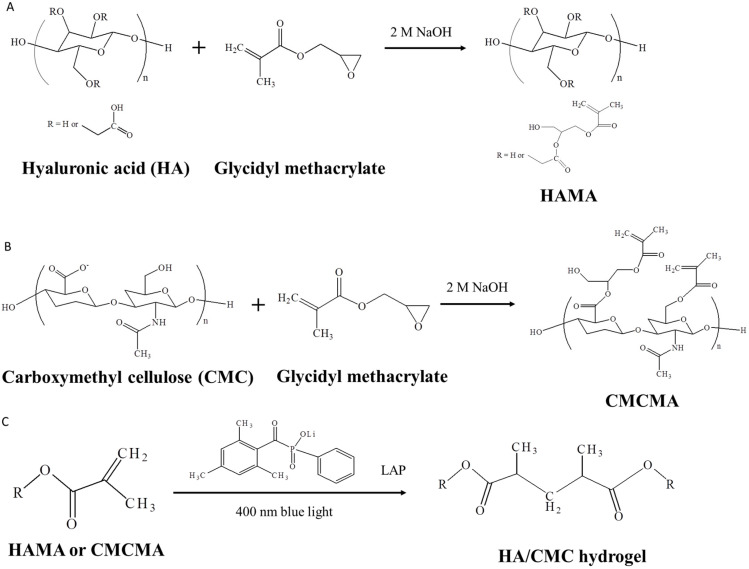
The synthesis of hyaluronic acid methacrylate (HAMA) (**A**), carboxymethyl cellulose methacrylate (CMCMA) (**B**) by reacting hyaluronic acid (HA) and carboxymethyl cellulose (CMC) with glycidyl methacrylate. (**C**) The synthesis of HA/CMC hydrogel by photo-crosslinking reactions.

The HAMA (or CMCMA) was prepared by reacting HA (or CMC) with glycidyl methacrylate (Figure 1A,B). By free radical polymerization between HAMA and CMCMA, using lithium phenyl-2,4,6-trimethylbenzoylphosphinate (LAP) as a light-inducible photo-initiator, the HA/CMC (HC) hydrogel could be formed through photo-crosslinking reactions between HAMA and CMCMA induced by 400 nm blue light (Figure 1C). The ^1^H NMR spectra of HA (or CMC) and HAMA (or CMCMA) support the successful introduction of methacrylate groups to both polymers (Figure S1). Using 1/3, 1/1 or 3/1 mass ratio of HAMA/CMCMA, HC13, HC11 or HC31 hydrogel was facilely prepared in this study.

**Figure 2:**
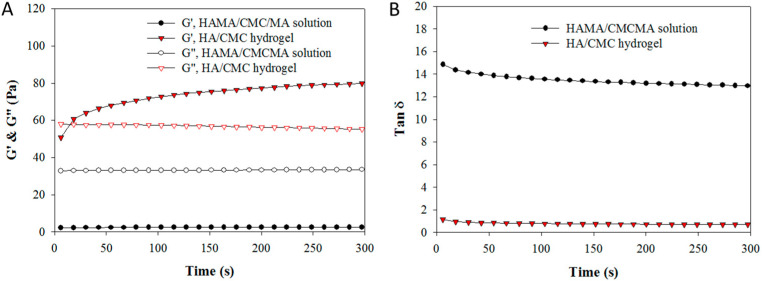
The storage modulus (G′) and loss modulus (G″) (**A**) and the loss tangent (tan δ) (**B**) of HAMA/CMCMA solution (1/3 mass ratio) and HA/CMC (HC13) hydrogel after photo-crosslinking.

The rheological analysis demonstrates that the storage modulus (G′) of HC13 hydrogel is much higher than that of the HAMA/CMCMA polymer solution (1/3 mass ratio), albeit the increase of loss modulus (G″) is less (Figure 2A). This results in a drastic reduction of the loss tangent (tan δ = G″/G′) of the polymer solution (~14) to the hydrogel (~1) after photo-crosslinking (Figure 2B). Taken together, these results indicate HA/CMC hydrogel could be synthesized from HAMA/CMCMA with blue light exposure for 3 min to induce sol-to-gel phase transition.

**Figure 3:**
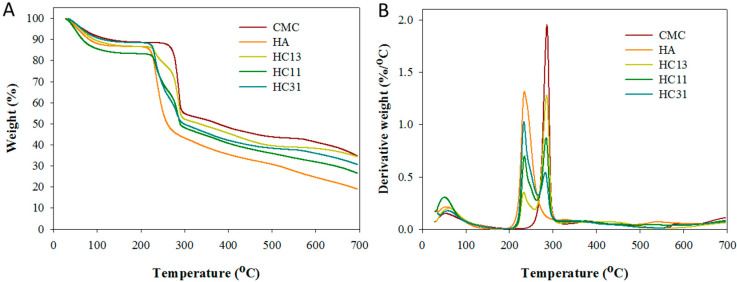
The thermogravimetric analysis (TGA) (**A**) and differential thermal gravimetric (DTG) analysis (**B**) of HA, CMC, and HA/CMC (HC) hydrogel prepared at 1/3 (HC13), 1/1 (HC11) and 3/1 (HC31) mass ratios of HAMA/CMCMA.

From thermogravimetric analysis (TGA), HA shows thermal decomposition starting at ~220 °C and the residual weight is 18.7% at 700 °C due to its natural polymer origin (Figure 3A). This results in a peak decomposition temperature at 234.5 °C from differential thermal gravimetric (DTG) analysis (Figure 3B). The other natural polymer CMC displays a higher peak decomposition temperature (285.9 °C) and a higher residual weight (35.1%) (Figure 3A). For all HA/CMC hydrogels, two peak decomposition temperatures, corresponding to HA and CMC, were observed regardless of the composition of the hydrogel (Figure 3B). This indicates the successful incorporation of HA and CMC in HC hydrogel by the photo-crosslinking reaction. The difference in the residual weight of different HC hydrogels (HC13 > HC11 > HC31), also correctly reflects the trend that a higher HAMA/CMCMA mass ratio during hydrogel preparation leads to lower CMC composition in the hydrogel (Figure 3A).

**Figure 4:**
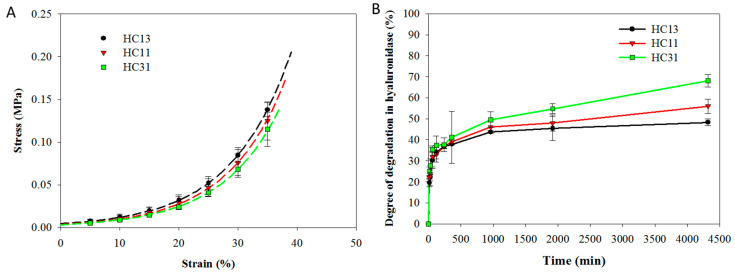
The typical stress-strain curves from unconfined compression tests (**A**) and the degradation in hyaluronidase (700 U/mL) solution (**B**) of HA/CMC hydrogel prepared at 1/3 (HC13), 1/1 (HC11) and 3/1 (HC31) mass ratios of HAMA/CMCMA.

The stress (σ)-strain (ε) data of HC13, HC11 and HC31 hydrogels from unconfined compression tests are fitted with the equation σ = Ae ^(Bε−1)^ and shown in Figure 4A. The ultimate stress and ultimate strain obtained from the compression tests, the compressive elastic modulus at different strain ratios, and the toughness of the hydrogel were analyzed and included in Table 1. In general, the elastic modulus increases with CMCMA composition in the hydrogel, which is also true for ultimate stress and toughness. In contrast, the ultimate strain is independent of hydrogel composition. This indicates the strength of the hydrogel is in the order HC13 > HC11 > HC31. Indeed, the failure stress increases by 42% from HC31 to HC13, while the toughness increases by 47.9%. Similarly, the compressive elastic modulus increased 15.5% from HC13 to HC31at ε = 0.3.

The biodegradation of HA/CMC hydrogel at 37 °C was studied in hyaluronidase solution, which is an enzyme that can break down HA in vivo. All hydrogels were rapidly degraded by hyaluronidase (700 U/mL) to 35% degree of degradation (DD) in 120 min (Figure 4B). The degradation rate gradually decreased, and the DD is dependent on composition of the hydrogel, which is 68.1% (HC31), 56.0% (HC11) and 48.3% (HC13) in 3 days. As CMC was not degraded by hyaluronidase, the increased DD correlates well with the increased weight percentage of HA in the composite hydrogel.

### 2.2. In Vitro Cell Culture

**Figure 5:**
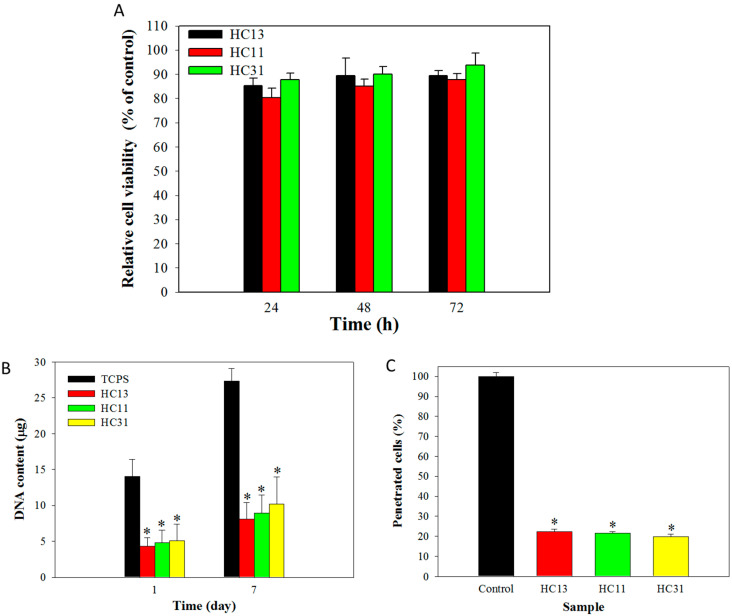
(**A**) The cytotoxicity of HA/CMC hydrogel prepared at 1/3 (HC13), 1/1 (HC11) and 3/1 (HC31) mass ratios of HAMA/CMCMA. The 3T3 fibroblasts were cultured with 24-h extract of hydrogel for 24, 48 and 72 h and fresh cell culture medium was used as a control to calculate the relative cell viability from WST-1 assay. (**B**) The DNA contents for 3T3 cells cultured on tissue culture polystyrene (TCPS) or HA/CMC hydrogel for 1 and 7 days. (**C**) The penetration of 3T3 cells by placing HA/CMC hydrogel in a cell insert of Transwell migration assay for determining the DNA content of penetrated cells in 24 h. The control is cell insert only. * *p* < 0.05 compared with TCPS or control.

The cytotoxicity of HA/CMC hydrogel was tested by culture 3T3 fibroblasts with a 24-h extract of the hydrogel at different times. The relative cell viability (normalized with fresh cell culture medium) from WST-1 cell proliferation and viability assay indicates all hydrogels are non-cytotoxic with higher than 80% relative cell viability (Figure 5A). From the cell attachment study with 3T3 fibroblasts, there is a significant reduction of attached cells to all hydrogel samples when compared with the control (tissue culture polystyrene, TCPS), where close to 70% reduction in cellular DNA content was found on day 1 (Figure 5B). Nonetheless, a similar cell proliferation rate was noted for cells on both TCPS and hydrogels, with increased DNA content from days 1–7. The cell proliferation rate, calculated from the DNA contents from day 1 to day 7, indicates no significant change in cell proliferation rate for cells grown on TCPS and on different hydrogels. Overall, the composition of hydrogel did not influence cell attachment or proliferation of attached cells. The cell penetration test, where the Transwell migration assay was used by fitting a hydrogel sample to a cell insert placed between dual chambers, indicates the percentage of 3T3 penetrating through the hydrogel is ~20% that of the control (cell insert without hydrogel) (Figure 5C). There is no significant difference found between hydrogel samples; nonetheless, all hydrogels can reduce the percentage of migrated (penetrated) cells. Overall, the in vitro cell culture study indicates that HA/CMC (HC) hydrogel can prevent penetration and reduce attachment of 3T3 fibroblasts, without inducing intrinsic cytotoxicity in free or attached cells.

**Figure 6:**
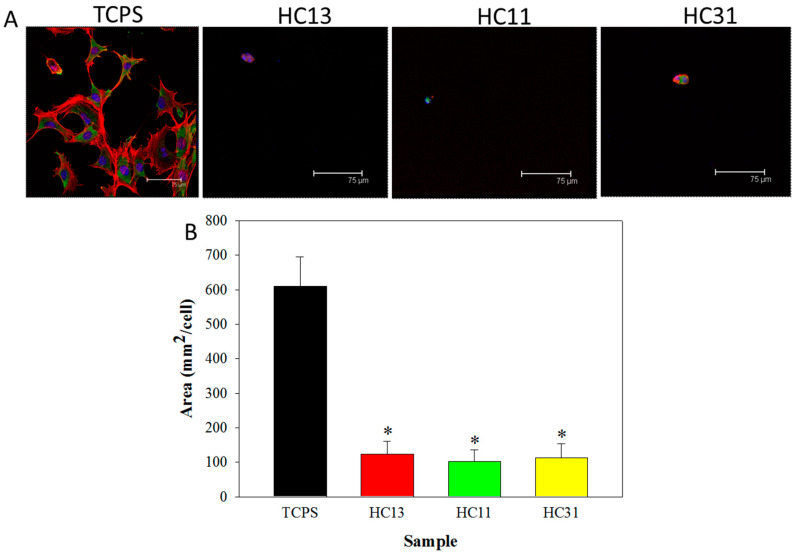
(**A**) The immunofluorescence (IF) staining image of vinculin (green), cytoskeleton (red) and nucleus (blue) after culture 3T3 fibroblasts on tissue culture polystyrene (TCPS) or HA/CMC hydrogel prepared at 1/3 (HC13), 1/1 (HC11) and 3/1 (HC31) mass ratios of HAMA/CMCMA. Bar = 75 μm. (**B**) The quantification of the vinculin expressing area from 5 high power field regions in the IF images. * *p* < 0.05 compared with TCPS.

The molecular mechanism to reduce cell attachment on hydrogel was studied from the expression of a focal adhesion protein (vinculin) and cytoskeletal actin in 3T3 fibroblasts. Fibroblasts cultured on TCPS or on hydrogel for 24 h were observed by a confocal microscope. From immunofluorescence (IF) staining of vinculin, the 3T3 cells expressed a high level of vinculin (green fluorescence) in the TCPS but significantly reduced expression of this focal adhesion-associated protein was noted for 3T3 cells on HC hydrogel surface (Figure 6A). Judging from the arrangement of red fluorescence cytoskeleton actin, round and small 3T3 cells were loosely attached to the hydrogel. Apparently, this is morphologically distinctive from that shown by cells on TCPS, which are flat, outstretching and display less cytoskeleton expression. Further quantification of the green fluorescence area percentage associated with vinculin indicates that fibroblasts on hydrogel express 17% of vinculin area compared with that on TCPS, with no difference found among different hydrogels, implicating HC hydrogel can prevent cell adhesion by reducing expression of focal adhesion protein (Figure 6B).

### 2.3. In Vivo Study

**Figure 7:**
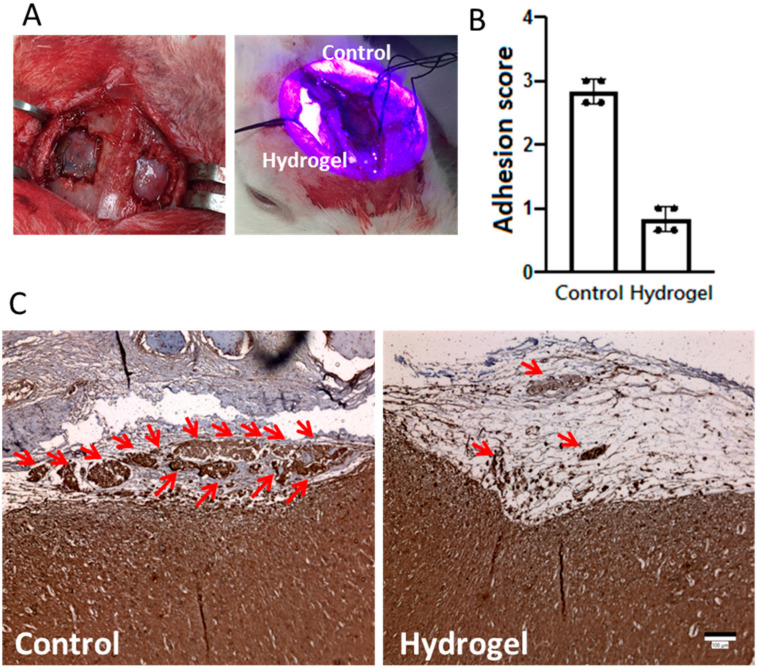
(**A**) A bilateral dural defects were created on the skull of rabbits and one defect was repaired with HC13 hydrogel (hydrogel) while the other was not treated (control). (**B**) The adhesion score from gross examination of the severity of post-surgical adhesion. 0: no adhesion; 1: mild adhesion; 2: moderate adhesion; 3: severe adhesion. (**C**) The immunohistochemical (IHC) staining of vimentin where red arrows indicate vimentin synthesized by fibroblasts in adhesion tissue. Bar = 100 μm.

Due to the best mechanical properties, comparable cellular response and anti-adhesion properties of HC13 hydrogel, this hydrogel was used for in vivo study with the rabbit dural defect model. The dural defect was created with skull trephination and a linear dura incision, and a small craniectomy was performed at both hemispheres to create a 0.5 × 0.5 cm defect (Figure 7A). One defect was covered fully with hydrogel solution and irradiated with a 400 nm blue light to seal the dura, while the other defect was not treated and used as a control (Figure 7A). There was no sign of infection post-operation for all animals receiving the treatments during the observation period. After four weeks, the rabbits were euthanized and the extent of adhesion at the surgical site was grossly examined. The severity of adhesion was scored by three independent observers who were blind to the procedures, with a score from 0 (no adhesion) to 3 (severe adhesion). As shown in Figure 7B, the null control group shows a significantly higher adhesion score than the hydrogel group, indicating HC13 hydrogel could be used as a dural substitute for preventive adhesion after craniotomy. The immunohistochemical (IHC) staining of vimentin shows a strong synthesis of vimentin in the epidural space in the control group (Figure 7C). In contrast, the hydrogel group shows a minimum stained intensity of vimentin within the residual hydrogel, implicating HC13 hydrogel can form a barrier in the filled dural defect space, to prevent migration of vimentin-expressing fibroblasts.

**Figure 8:**
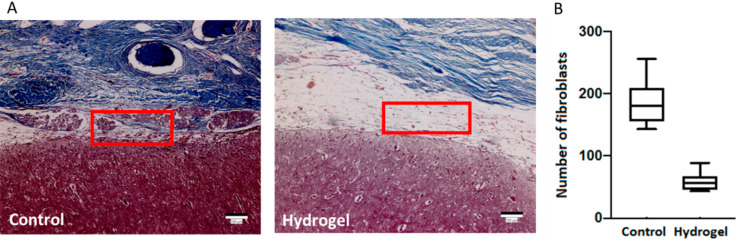
(**A**) The hematoxylin-eosin (H&E) staining image of untreated dural defect (control) and dural defect treated HC13 hydrogel (hydrogel). (**B**) The number of fibroblasts in the formed adhesion tissue between brain and muscle layer of scalp (circled in red) was determined from direct cell count under high power field.

The extent of post-operative adhesion was further determined from H&E stain. Severe adhesions between brain and muscle layer of the scalp were found in untreated control group. In contrast, excellent anti-adhesion effect was found for the hydrogel-treated group, with minimum loose fibrous tissue formation and maintenance of sizable interval between brain and muscle layer of scalp (Figure 8A). The extent of adhesion from histological observation was further confirmed from direct count of fibroblasts within the red-circled adhesion tissue in the H&E stain images under high power field. As shown in Figure 8B, the HC13 hydrogel can significantly lower the fibroblast counts compared with the control group. As adhesions are reported to be formed by proliferating tissue-resident fibroblasts, the HC13 hydrogel is deemed to be with anti-adhesion effect by acting as a dural substitute to prevent direct contact between brain and fascia layer [39].

## 3. Discussion

Among HA derivatives, the derivative where methacrylate groups are attached to the polymer backbone (hyaluronic acid methacrylate, HAMA) is attractive for making hydrogels (Figure 1A). This stems from their easy one-step synthesis, good biocompatibility and photo-crosslinkable ability in the presence of a suitable photo-initiator [40]. To prepare HA/CMC hydrogel, we adopt a similar strategy to synthesize CMCMA from CMC by reacting CMC with glycidyl methacrylate in an alkaline solution (Figure 1B). By modifying the HA and CMC with methacrylate groups, HAMA and CMCMA were used as base matrices for the development of photo-crosslinked HA/CMC hydrogel (Figure 1C). With LAP as a photo-initiator, crosslinked HA/CMC copolymer hydrogel network was formed by exposing HAMA/CMCMA/LAP solution to blue light as in stereolithographic bioprinting [41]. The HAMA and CMCHA were successively synthesized as demonstrated by NMR analysis (Figure S1, supplementary materials). Regardless of the composition of the hydrogel, where 25% (*w*/*w*), 50% (*w*/*w*) or 75% (*w*/*w*) HAMA was used, hydrogel formation was completed within 3 min after free-radical photo-crosslinking reactions induced by 400 nm blue light. From the rheological analysis of HAMA/CMCMA polymer solution and HC hydrogel, a sharp increase in storage modulus (G′), the energy stored in the elastic structure of the sample, was observed for the hydrogel over the polymer solution (Figure 2A). This increased storage modulus indicates effective intramolecular crosslinking between HAMA and CMCMC to HA/CMC copolymer chains, which can provide more structure within the material [42]. On the other hand, the loss modulus (G″), the amount of energy dissipated in the sample, showed less change. Overall, the hydrogel was more elastic than the polymer solution after gelation, with a sharp decrease of loss tangent (tan δ), supporting successful hydrogel formation by exposing HAMA/CMCMA polymer solution to blue light exposure for 3 min (Figure 2B) [43].

The influence of HA composition on mechanical properties and thermal decomposition behavior was studied. Overall, both HA and CMC were found within the HC hydrogel from TGA/DTG analysis and the residual weight is consistent with the weight percentage of each component in the composite hydrogel (Figure 3). As commonly employed for photo-crosslinked hydrogels, unconfined compression testing was used to determine the mechanical properties of hydrogel [44]. It should be noted that the hydrated hydrogel is too weak for tensile testing in the study. At the highest CMC content, the HC13 hydrogel displays the best mechanical properties (Figure 4A). With the increasing weight percentage of CMC in HC hydrogel, its mechanical properties improved, as shown by the higher compression strength, elastic modulus and toughness (Table 1). Overall, this is similar to the effect of CMC on the mechanical properties of blended CMC/carbohydrate (starch), although a similar shift of peak decomposition temperature was not observed from the DTG analysis [45]. As biodegradation ability is essential for implant biomaterial as a dural substitute, HC hydrogels are demonstrated to be biodegradable in hyaluronidase solution at body temperature (Figure 4B). The degree of degradation (DD) coincides with the weight percentage of HA in the composite hydrogel from the non-degradable nature of CMC by hyaluronidase. The DD of HC31 hydrogel is 70% in 3 days, similar to HA hydrogel used in the peritoneal cavity [46].

The possible cytotoxicity of HC hydrogel was determined by culturing fibroblasts with the 24-h extract of different hydrogel samples, to realize whether components in hydrogels show any cytotoxic effects. By normalizing the cell viability of the sample with that of the control (fresh cell culture medium), the relative cell viability of all hydrogels is above 70%, indicating minimum cytotoxicity to meet the requirement for a non-toxic medical device (ISO 10993-5 standard) (Figure 5A). To examine the attachment and proliferation of fibroblasts, 3T3 cells were seeded on TCPS or hydrogel surface and cultured for up to 7 days. A comparison of the DNA content on day 1 indicates a 70% reduction in the attached cell number on the hydrogel from TCPS (Figure 5B). This is consistent with previous findings that HA can inhibit cell attachment [47]. For cell proliferation, the DNA contents on day 7 follow the same trend as day 1, with TCPS showing the highest cell number than hydrogel (Figure 5B). After normalizing the DNA content at 7 with its respective value at day 1 for all groups, the increase in cell number based on DNA contents was found to be not significantly different among TCPS and hydrogels. Therefore, the hydrogel only reduces cell attachment but not cell proliferation, which is beneficial for tissue healing. Considering the influence of each component in the hydrogel, information regarding CMC on fibroblast attachment and proliferation is limited. For HA, an early study indicated that surface-linked HA can prevent fibroblast cell adhesion in vitro [48]. The adhesion of fetal fibroblasts was reported to strongly depend on the delivery method of HA, as well as the concentration and molecular weight of delivered HA [49]. By increasing the concentration and molecular weight of HA in HA-containing hydrogels, fibroblasts showed reduced cell adhesion with a more rounded cell shape [50]. The less cell spreading of attached dermal fibroblasts to HA-modified surfaces was found to be mediated primarily by the HA receptor CD44 from immunofluorescence microscopy observations [51]. The adhesion of fibroblasts to silicone rubber was significantly inhibited by coating with HA in vitro, as well as with less fibrinogen adsorption [52].

The penetration and migration of fibroblasts is a major cause of adhesion formation. Therefore, a Transwell cell migration assay was used to determine cell penetration through a cell culture insert to the lower well in a dual-chamber cell culture plate after 24 h. With imposed serum concentration gradient between chambers, fibroblasts in the upper chamber will tend to penetrate through the hydrogel placed in the cell insert and move down to the bottom well. From cellular DNA contents of penetrated cells in the lower chamber, significantly fewer penetrated cells were found when the hydrogel was used (Figure 5C). The number of cells dropped to below 20% of the value shown by the control, which did not have hydrogel in the cell insert. The hindering mechanism could be explained by the barrier effect of the hydrogel, which can prevent penetration of fibroblastic cells, which can contribute to adhesion formation. The attachment of fibroblasts to the barrier is the first event responsible for adhesion formation; therefore, further study of the expression of a focal adhesion protein (vinculin) in fibroblasts on different substrates was used to proof the molecular mechanism responsible for the reduced cell attachment [53]. The cytoskeletal actin distribution and the expression of vinculin in fibroblasts were examined by immunofluorescence staining (Figure 6A). When cultured on TCPS, fibroblasts demonstrated increased cellular spreading, well-distributed actin cytoskeleton and enhanced vinculin expression. In contrast, when the cells were cultured on hydrogels, they maintained a rounded morphology, diffused actin cytoskeletal distribution and minimal vinculin expression (Figure 6B).

Post-operative adhesions involve complex mechanisms that are yet to be fully understood, which makes the development of safe and effective therapeutic options to mitigate post-operative adhesion formation to be a continued unmet medical challenge [54]. An animal skull defect model is standard to test a dural substitute, therefore, dural defect models from rabbits were used in this study (Figure 7A) [55]. In the in vivo study, less adhesion is observed compared to the null control from gross observation as the hydrogel can act as a barrier between brain and muscle fascia (Figure 7B). As vimentin is an adhesion molecule useful for assessing endothelial sprouting, this marker protein was used to assess the adhesion reduction from the action of hydrogel [56]. From IHC staining of the vimentin in the adhesion tissue, less vimentin was synthesized by fibroblasts (Figure 7C), due to reduced fibroblast attachment and penetration in vitro. Considering vimentin, this intermediate filament protein plays an important role in cell adhesion and spreading, like the actin cytoskeleton [57]. Vimentin is a widely recognized phenotypic marker for identifying fibroblasts [58], while recent studies indicate its key role in adhesion is by regulating integrin functions [59]. By contributing to the mechanical stabilization of cell structure, the expression of vimentin is crucial for effective tissue regeneration and wound healing [60]. It also helps to control the assembly of cell adhesions and migration through collagen matrices, while higher vimentin expression is associated with enhanced cell adhesion to the extracellular matrix and collagen deposition [61].

The adhesion and spreading of fibroblasts are critical during wound healing and tissue remodeling, from which post-operative adhesion formation was linked to proliferating tissue-resident fibroblasts [39]. From in vivo study, the direct observation of fibroblasts in the formed adhesion tissue between the brain and muscle layers of the scalp indicates a drastic reduction of the resident fibroblast population in the hydrogel group from the control group (Figure 8). Indeed, by preventing the penetration of fibroblasts, the application of HC13 represents an effective approach to reducing adhesion at repaired dura site, where fibroblasts originating from the muscle layer of the scalp are prone to form peridural fibrosis after craniectomy. Another role played by HA in HC hydrogel may be the reduction of the local inflammatory response induced by surgical trauma, which is an important feature of potential pathologic adhesion formation [62]. It was reported that HA can interfere with inflammation-promoting compounds, such as cytokines and prostaglandins [63]. The anti-inflammatory effect of HA to reduce the production of pro-inflammatory cytokines was postulated to be related to the interaction of HA with CD44 as its receptor [64]. Since the reduction of the initial inflammatory reaction could be a possible way to reduce fibrosis, drug-eluting HA/CMC hydrogel could be designed in the future by mixing an anti-inflammatory agent with HAMA/CMCHA solution in the liquid state. This could further expand the versatility of HC hydrogel for controlled drug delivery after its formation in situ. For instance, ibuprofen as an anti-inflammatory agent has been combined with a light-curable furfuryl hyaluronic acid derivative for anti-adhesion applications [65].

## 4. Materials and Methods

### 4.1. Materials

Sodium salt of hyaluronic acid (HA, average molecular weight = 1.3 MDa) was obtained from Bloomage Freda Biopharm Co. Ltd. (Jinan, Shandong, China). Sodium salt of carboxymethyl cellulose (CMC, high viscosity, degree of substitution = 0.89), Dulbecco’s Modified Eagle’s medium (DMEM), DNA quantitation kit for fluorescence assay and WST-1 assay kit for cell proliferation and viability were obtained from Sigma-Aldrich (St Louis, MO, USA). DuraGen was obtained from Integra Life Sciences (Princeton, NJ, USA).

### 4.2. Preparation and Characterization of HAMA and CMCMA

To prepare HAMA and CMCMA, 25 g of HA or CMC was dissolved in 450 mL of 2 M NaOH solution at 30 °C and 50 °C, respectively. Fifty milliliters of glycidyl methacrylate were added to the HA solution and incubated at 4 °C for 72 h. After adding acetone to induce precipitation, HAMA was recovered by centrifugation for 20 min at 5000× *g*. The precipitate was recovered and air-dried at 30 °C, followed by dialyzing against distilled water (DI) for 72 h and freeze-drying. For CMCMA, 100 mL of glycidyl methacrylate was added to 400 mL CMC and incubated at 50 °C for 72 h. Following the same purification method using acetone precipitation, CMCMA was collected in the precipitate and air-dried. The air-dried CMCMA was dialyzed against DI water for 72 h and freeze-dried. For nuclear magnetic resonance (NMR) analysis, HAMA or CMCMA was dissolved in D_2_O to obtain ^1^H NMR spectra with Bruker Avance III HD 600 MHz NMR spectrometer (Billerica, MA, USA).

### 4.3. Preparation of HA/CMC (HC) Hydrogel

Different mass ratios of HAMA/CMCMA were used to prepare HA/CMC hydrogel with suitable gel-forming properties and stability. For preparing HA/CMC hydrogel with 1/3 mass ratio (HC13), 0.5% (*w*/*w*) HAMA and 1.5% (*w*/*w*) CMCMA were prepared in phosphate-buffered saline (PBS). An aliquot (10 μL) of the photo-initiator stock solution (lithium phenyl-2,4,6-trimethylbenzoylphosphinate, LAP, 500 mg/mL) was added to 1 mL of the polymer precursor solution. The solution was irradiated with blue light (400 nm, 1.5 mW/cm^2^) for 3 min to induce photo-crosslinking. For HA/CMC hydrogels with 1/1 (HC11) or 3/1 (HC31) mass ratios, similar protocol follows, by using 1% (*w*/*w*) HAMA/1% (*w*/*w*) CMCMA or 1.5% (*w*/*w*) HAMA/0.5% (*w*/*w*) CMCMA. For cell culture studies, the hydrogel solution was sterile by filtering through a 0.45 μm filter before gelation.

### 4.4. Rheological Analysis of Polymer Solution and Hydrogel

The rheological properties of HAMA/CMCMA polymer solution (1/3 mass ratio) and HA/CMC hydrogel (HC13) were determined using a Discovery HR-2 Hybrid Rheometer (TA Instruments, New Castle, DE, USA) at 37 °C. A 60 mm diameter cone-and-plate with a 2-degree cone angle was used. The oscillation step model with frequency = 1 Hz, oscillation = 0.1 rad was used to obtain the storage modulus (G′), loss modulus (G″) and loss tangent (tan δ) using the Rheology Solutions software from TA Instruments.

### 4.5. Mechanical Properties Analysis

To investigate the mechanical properties of the hydrogel, unconfined compression tests were performed for different hydrogel samples using ElectroForce 5200 BioDynamic Test Instrument (Bose, Eden Prairie, MN, USA). The hydrogels were soaked in PBS for 24 h prior to testing. A 250 N compression load was applied at a crosshead speed of 0.02 mm/s. The stress (σ)-strain (ε) data were recorded when subject to uniaxial stress. The ultimate stress and ultimate strain values were defined as the point where failure of the hydrogel occurred. The stress-strain data up to failure was fitted with a non-linear equation, σ = Ae ^(Bε−1)^, where A and B are fitting constants. The elastic modulus at 10%, 20% or 30% strain was calculated with this equation from the slope of the tangent to the stress-strain curve. The toughness, defined as the required energy to deform a sample to failure, was obtained from the area under the stress-strain curve.

### 4.6. Thermogravimetric Analysis (TGA) and Differential Thermal Gravimetric (DTG) Analysis

To examine the thermal stability of the hydrogel, TGA and DTG analyses were carried out from 35–700 °C (10 °C/min) using TGA 2050 (TA instruments, New Castle, DE, USA) under an inert nitrogen atmosphere.

### 4.7. In Vitro Degradation

A pre-weighed hydrogel was immersed in a hyaluronidase solution (700 U/mL) and the rate of degradation was measured. The sample was removed from the solution at different times and its residual weight was determined after drying. The degree of degradation (DD) was calculated from, DD (%) = 100 × (m_i_ − m_t_)/m_i_, where m_i_ and m_t_ are the initial weight of hydrogel and the weight at time t, respectively. Each measurement was repeated five times.

### 4.8. In Vitro Cell Culture

In order to determine the cytotoxicity of the hydrogel, the WST-1 assay for cell proliferation and viability was used. Briefly, ~0.5 g hydrogel was immersed in 500 μL of cell culture medium (DMEM medium supplemented with 10% fetal bovine serum, 1% penicillin–streptomycin) at 37 °C for 24 h. The extract was added to each well in a 96-well culture plate containing mouse fibroblastic NIH 3T3 cells (5 × 10^3^/well) and incubated at 37 °C in a humidified atmosphere containing 5% CO_2_. After being cultured for 24, 48 and 72 h, the culture medium was removed from the well, followed by gently washing cells with 0.1 M PBS (pH 7.4). An aliquot of 10 μL of WST-1 reagent was added to each well and the plate was incubated for 4 h at 37 °C and 5% CO_2_. A microplate reader was used to measure the solution absorbance at 450 nm. The control is cells cultured with a fresh cell culture medium.

For cell adhesion assay, 3T3 cells were seeded directly onto the hydrogel and compared with cells seeded on tissue culture polystyrene (TCPS). After culture in cell culture medium at 37 °C for 1 and 7 days, cell number was determined from DNA assays. For immunofluorescence (IF) staining of the focal adhesion protein vinculin, 3T3 cells cultured on hydrogel and TCPS were stained with anti-vinculin primary antibody (Abcam, Cambridge, UK) and FITC-AffiniPure goat anti-mouse IgG secondary antibody (Jackson ImmunoResearch, West Grove, PA, USA). The cytoskeletal organization and nucleus were visualized with TRITC-conjugated Phalloidin and Hoechst 33528, respectively. After staining, cells were observed under a Zeiss LSM 510 Meta inverted confocal laser scanning microscope (Jena, Germany) for red (actin cytoskeleton), green (vinculin) and blue (nucleus) fluorescence.

For cell penetration assay, a double chamber dish separated by a cell culture insert was used to evaluate the barrier effect of hydrogel in vitro. Mouse fibroblastic NIH 3T3 cells (1 × 10^4^ cells) were placed in the upper chamber containing DMEM with 2% fetal bovine serum (FBS) and the lower chamber was filled with DMEM with 10% FBS. A hydrogel sample was placed at the bottom of the insert to determine cell migration induced by serum gradient to the lower chamber in 24 h at 37 °C, [25]. The control is using insert alone without hydrogel. The cells in the lower chamber were determined with a DNA quantitation kit.

### 4.9. In Vivo Dural Defect Animal Model

All animal experiment procedures performed in this study were approved by the Institutional Animal Care and Use Committee (IACUC) of Chang Gung University (IACUC approval number CGU107-271). New Zealand White rabbits (age > 10 weeks) were selected for the dural substitute study (n = 4). Dura was wide-opened after bilateral craniotomy procedures in the animals, which were anesthetized with Zoletil (10 mg/kg) and Xylazine (5 mg/kg). In brief, a linear incision was made along the scalp and a small craniectomy was performed at both hemispheres in ~0.5 × 0.5 cm size. The dura is cut and removed as extensively as possible. An HC13 hydrogel solution containing 500 mg/mL LAP was employed to fully cover one of the dural defects, followed by irradiating the defect with 400 nm blue light at 1.5 mW/cm^2^ for 3 min. The dura was sealed with the HC31 hydrogel immediately. The other dural defect was not treated and used as a control. All animals underwent regular body weight measurements and examinations for neurotoxicity, wound infection and CSF leakage. After 4 weeks, the rabbits were euthanized, and the surgical site was grossly examined by three independent observers who were blind to the procedures for the extent of post-operative dural adhesion. The adhesion grading scale is from 0–3; 0 indicates dura is free from adhesion; 1 indicates a thin fibrous band formation; 2 indicates more than 1 mm of fibrous tissue formation; 3 indicates severe scarring. The surgical site of the dural defect (including the surrounding skull) was removed for decalcification and fixation, followed by paraffin embedding. The embedded tissue was cut into 8-μm thickness for histological examination from H&E staining and vimentin immunohistochemical (IHC) staining.

### 4.10. Statistical Analysis

The data are presented as mean ± standard deviation. Statistical significance was declared when the p value was less than 0.05. One-way analysis of variance (ANOVA) was used for statistical analysis between groups.

## 5. Conclusions

In this study, photo-crosslinked hydrogels were successfully developed from HA methacrylate (HAMA) and CMC methacrylate (HAMA). With short-term blue light exposure for photo-crosslinking, light-responsive HAMA and CMCMA were crosslinked to form a hydrogel in situ for dural repair. The HA/CMC hydrogel is biocompatible, bio-degradable, and mechanically robust. It also shows a drastic reduction of cellular attachment and penetration of adhesion-forming fibroblasts in vitro, implicating the anti-adhesion effect in vivo. From in vivo experiments, the HC13 hydrogel can function as a dural substitute to repair dural defects in rabbits. In addition, the hydrogel can substantially reduce dural adhesion in 4 weeks when compared with the null control. Taken together, a novel dural substitute is proposed in this study, which can be endowed with new features to meet the need in difficult dural repair conditions.

## Figures and Tables

**Table 1:** The mechanical properties from unconfined compression tests of HA/CMC hydrogel prepared at 1/3 (HC13), 1/1 (HC11) and 3/1 (HC31) mass ratios of HAMA/CMCMA.

Properties	HC13	HC11^+^	HC31
Compressive elastic modulus at ε = 0.1 (MPa)	0.12 ± 0.02 ^#^	0.10 ± 0.03	0.09 ± 0.01
Compressive elastic modulus at ε = 0.2 (MPa)	0.31 ± 0.03 ^#^	0.28 ± 0.06	0.25 ± 0.03
Compressive elastic modulus at ε = 0.3 (MPa)	0.82 ± 0.01 *^,#^	0.75 ± 0.03	0.71 ± 0.05
Compressive strain to failure, ε_max_ (%)	39.0 ± 1.7	38.3 ± 1.5	37.5 ± 0.7
Compressive stress to failure, σ_max_ (MPa)	0.20 ± 0.04 ^#^	0.17 ± 0.05	0.14 ± 0.02
Toughness (kJ/m^3^)	20.7 ± 1.40 *^,#^	17.5 ± 1.06 ^#^	14.0 ± 0.75

* *p* < 0.05 compared with HC11, ^#^ *p* < 0.05 compared with HC31.

## Data Availability

The data presented in this study are available on request from the corresponding author.

## Supplementary Materials

Supplementary Materials are available online at https://www.mdpi.com/article/10.3390/ijms23116177/s1.

## References

[1] Berjano R., Vinas F.C., Dujovny M. (1999). A review of dural substitutes used in neurosurgery. Crit. Rev. Neurosurg..

[2] Martínez-Lage J.F., Pérez-Espejo M.A., Palazón J.H., Hernández F.L., Puerta P. (2006). Autologous tissues for dural grafting in children: A report of 56 cases. Childs Nerv. Syst..

[3] Choi J.S., Chang S.J. (2018). A Comparison of the Incidence of Post-Dural Puncture Headache and Backache After Spinal Anesthesia: A Pragmatic Randomized Controlled Trial. Worldviews Evid. Based Nurs..

[4] Feng C., Qianqian S., Jianhua H., Yu Z., Yipeng W., Jianguo Z., Guixing Q. (2019). Treatment experience for full-thickness wound dehiscence with cerebrospinal fluid leakage following posterior primary spine surgery: Three case reports. Medicine.

[5] MacEwan M.R., Kovacs T., Osbun J., Ray W.Z. (2018). Comparative analysis of a fully-synthetic nanofabricated dura substitute and bovine collagen dura substitute in a large animal model of dural repair. Interdiscip. Neurosurg..

[6] Parízek J., Mĕricka P., Spacek J., Nĕmecek S., Eliás P., Sercl M. (1989). Xenogeneic pericardium as a dural substitute in reconstruction of suboccipital dura mater in children. J. Neurosurg..

[7] Deng K., Ye X., Yang Y., Liu M., Ayyad A., Zhao Y., Yuan Y., Zhao J., Xu T. (2016). Evaluation of efficacy and biocompatibility of a new absorbable synthetic substitute as a dural onlay graft in a large animal model. Neurol. Res..

[8] Mukai T., Shirahama N., Tominaga B., Ohno K., Koyama Y., Takakuda K. (2008). Development of watertight and bioabsorbable synthetic dural substitutes. Artif. Organs.

[9] Yamada K., Miyamoto S., Nagata I., Kikuchi H., Ikada Y., Iwata H., Yamamoto K. (1997). Development of a dural substitute from synthetic bioabsorbable polymers. J. Neurosurg..

[10] Barbolt T.A., Odin M., Léger M., Kangas L., Hoiste J., Liu S.H. (2001). Biocompatibility evaluation of dura mater substitutes in an animal model. Neurol. Res..

[11] Pohlenz P., Adler W., Li L., Schmelzle R., Klatt J. (2013). Medial orbital wall reconstruction with flexible Ethisorb^®^ patches. Clin. Oral Investig..

[12] Cohen A.R., Aleksic S., Ransohoff J. (1989). Inflammatory reaction to synthetic dural substitute. Case report. J. Neurosurg..

[13] Vakis A., Koutentakis D., Karabetsos D., Kalostos G. (2006). Use of polytetrafluoroethylene dural substitute as adhesion preventive material during craniectomies. Clin. Neurol. Neurosurg..

[14] Bouten P.J.M., Zonjee M., Bender J., Yauw S.T.K., van Goor H., van Hest J.C.M., Hoogenboom R. (2014). The chemistry of tissue adhesive materials. Prog. Polym. Sci..

[15] Wright N.M., Park J., Tew J.M., Kim K.D., Shaffrey M.E., Cheng J., Choudhri H., Krishnaney A.A., Graham R.S., Mendel E. (2015). Spinal sealant system provides better intraoperative watertight closure than standard of care during spinal surgery: A prospective, multicenter, randomized controlled study. Spine.

[16] Zhu T., Wang H., Jing Z., Fan D., Liu Z., Wang X., Tian Y. (2022). High efficacy of tetra-PEG hydrogel sealants for sutureless dural closure. Bioact. Mater..

[17] Sabatino G., Della Pepa G.M., Bianchi F., Capone G., Rigante L., Albanese A., Maira G., Marchese E. (2014). Autologous dural substitutes: A prospective study. Clin. Neurol. Neurosurg..

[18] Nunamaker E.A., Kipke D.R. (2010). An alginate hydrogel dura mater replacement for use with intracortical electrodes. J. Biomed. Mater. Res. B Appl. Biomater..

[19] Hadaegh A., Burns J., Burgess L., Rose R., Rowe E., LaMorte W.W., Becker J.M. (1997). Effects of hyaluronic acid/carboxymethylcellulose gel on bowel anastomoses in the New Zealand white rabbit. J. Gastrointest. Surg..

[20] Isık S., Taşkapılıoğlu M., Atalay F.O., Dogan S. (2015). Effects of cross-linked high-molecular-weight hyaluronic acid on epidural fibrosis: Experimental study. J. Neurosurg. Spine.

[21] He Y., Revel M., Loty B. (1995). A quantitative model of post-laminectomy scar formation. Effects of a nonsteroidal anti-inflammatory drug. Spine.

[22] Li L., Wang N., Jin X., Deng R., Nie S., Sun L., Wu Q., Wei Y., Gong C. (2014). Biodegradable and injectable in situ cross-linking chitosan-hyaluronic acid based hydrogels for postoperative adhesion prevention. Biomaterials.

[23] Diamond M.P., Burns E.L., Accomando B., Mian S., Holmdahl L. (2012). Seprafilm^®^ adhesion barrier: (2) a review of the clinical literature on intraabdominal use. Gynecol. Surg..

[24] Chen S.H., Chen C.H., Shalumon K.T., Chen J.P. (2014). Preparation and characterization of antiadhesion barrier film from hyaluronic acid-grafted electrospun poly(caprolactone) nanofibrous membranes for prevention of flexor tendon postoperative peritendinous adhesion. Int. J. Nanomed..

[25] Chou P.-Y., Chen S.-H., Chen C.-H., Chen S.-H., Fong Y.T., Chen J.-P. (2017). Thermo-responsive in-situ forming hydrogels as barriers to prevent post-operative peritendinous adhesion. Acta Biomater..

[26] Hu M.H., Yang K.C., Sun Y.H., Chen Y.C., Yang S.H., Lin F.H. (2017). In situ forming oxidised hyaluronic acid/adipic acid dihydrazide hydrogel for prevention of epidural fibrosis after laminectomy. Eur. Cells Mater..

[27] Hagberg L., Gerdin B. (1992). Sodium hyaluronate as an adjunct in adhesion prevention after flexor tendon surgery in rabbits. J. Hand Surg..

[28] An J.M., Shahriar S.M.S., Hasan M.N., Cho S., Lee Y.-K. (2021). Carboxymethyl Cellulose, Pluronic, and Pullulan-Based Compositions Efficiently Enhance Antiadhesion and Tissue Regeneration Properties without Using Any Drug Molecules. ACS Appl. Mater. Interfaces.

[29] Jeong D., Joo S.-W., Hu Y., Shinde V.V., Cho E., Jung S. (2018). Carboxymethyl cellulose-based superabsorbent hydrogels containing carboxymehtyl β-cyclodextrin for enhanced mechanical strength and effective drug delivery. Eur. Polym. J..

[30] Swilem A.E., Oyama T.G., Oyama K., Kimura A., Taguchi M. (2022). Development of carboxymethyl cellulose/gelatin hybrid hydrogels via radiation-induced cross-linking as novel anti-adhesion barriers. Polym. Degrad. Stab..

[31] Sannino A., Demitri C., Madaghiele M. (2009). Biodegradable Cellulose-based Hydrogels: Design and Applications. Materials.

[32] Berdah S.V., Mariette C., Denet C., Panis Y., Laurent C., Cotte E., Huten N., Le Peillet E.F., Duron J.J. (2014). A multicentre, randomised, controlled trial to assess the safety, ease of use, and reliability of hyaluronic acid/carboxymethylcellulose powder adhesion barrier versus no barrier in colorectal laparoscopic surgery. Trials.

[33] Diamond M.P., Burns E.L., Accomando B., Mian S., Holmdahl L. (2012). Seprafilm^®^ adhesion barrier: (1) a review of preclinical, animal, and human investigational studies. Gynecol. Surg..

[34] Kim J., Lee J.-H., Yoon J.-H., Chang J., Bae J., Kim K.-S. (2007). Antiadhesive Effect of the Mixed Solution of Sodium Hyaluronate and Sodium Carboxymethylcellulose after Endoscopic Sinus Surgery. Am. J. Rhinol..

[35] Beck D.E., Cohen Z., Fleshman J.W., Kaufman H.S., van Goor H., Wolff B.G. (2003). A Prospective, Randomized, Multicenter, Controlled Study of the Safety of Seprafilm^®^ Adhesion Barrier in Abdominopelvic Surgery of the Intestine. Dis. Colon Rectum.

[36] Takeuchi H., Kitade M., Kikuchi I., Shimanuki H., Kinoshita K. (2006). A novel instrument and technique for using Seprafilm hyaluronic acid/carboxymethylcellulose membrane during laparoscopic myomectomy. J. Laparoendosc. Adv. Surg. Tech. A.

[37] Mumert M.L., Altay T., Couldwell W.T. (2012). Technique for decompressive craniectomy using Seprafilm as a dural substitute and anti-adhesion barrier. J. Clin. Neurosci..

[38] Potts T.V., Petrou A. (1990). Laser photopolymerization of dental materials with potential endodontic applications. J. Endod..

[39] Foster D.S., Marshall C.D., Gulati G.S., Chinta M.S., Nguyen A., Salhotra A., Jones R.E., Burcham A., Lerbs T., Cui L. (2020). Elucidating the fundamental fibrotic processes driving abdominal adhesion formation. Nat. Commun..

[40] Schuurmans C.C.L., Mihajlovic M., Hiemstra C., Ito K., Hennink W.E., Vermonden T. (2021). Hyaluronic acid and chondroitin sulfate (meth)acrylate-based hydrogels for tissue engineering: Synthesis, characteristics and pre-clinical evaluation. Biomaterials.

[41] Lam T., Dehne T., Krüger J.P., Hondke S., Endres M., Thomas A., Lauster R., Sittinger M., Kloke L. (2019). Photopolymerizable gelatin and hyaluronic acid for stereolithographic 3D bioprinting of tissue-engineered cartilage. J. Biomed. Mater. Res. B Appl. Biomater..

[42] Velasco-Rodriguez B., Diaz-Vidal T., Rosales-Rivera L.C., García-González C.A., Alvarez-Lorenzo C., Al-Modlej A., Domínguez-Arca V., Prieto G., Barbosa S., Martínez J.F.A.S. (2021). Hybrid Methacrylated Gelatin and Hyaluronic Acid Hydrogel Scaffolds. Preparation and Systematic Characterization for Prospective Tissue Engineering Applications. Int. J. Mol. Sci..

[43] Poldervaart M.T., Goversen B., de Ruijter M., Abbadessa A., Melchels F.P.W., Öner F.C., Dhert W.J.A., Vermonden T., Alblas J. (2017). 3D bioprinting of methacrylated hyaluronic acid (MeHA) hydrogel with intrinsic osteogenicity. PLoS ONE.

[44] Fenn S.L., Oldinski R.A. (2016). Visible light crosslinking of methacrylated hyaluronan hydrogels for injectable tissue repair. J. Biomed. Mater. Res. Part B Appl. Biomater..

[45] Tavares K.M., Campos A.D., Luchesi B.R., Resende A.A., Oliveira J.E.d., Marconcini J.M. (2020). Effect of carboxymethyl cellulose concentration on mechanical and water vapor barrier properties of corn starch films. Carbohydr. Polym..

[46] Sakai S., Ueda K., Taya M. (2015). Peritoneal adhesion prevention by a biodegradable hyaluronic acid-based hydrogel formed in situ through a cascade enzyme reaction initiated by contact with body fluid on tissue surfaces. Acta Biomater..

[47] Sheehan K.M., DeLott L.B., West R.A., Bonnema J.D., DeHeer D.H. (2004). Hyaluronic acid of high molecular weight inhibits proliferation and induces cell death in U937 macrophage cells. Life Sci..

[48] Cassinelli C., Morra M., Pavesio A., Renier D. (2000). Evaluation of interfacial properties of hyaluronan coated poly(methylmethacrylate) intraocular lenses. J. Biomater. Sci. Polym. Ed..

[49] Morra M. (2005). Engineering of Biomaterials Surfaces by Hyaluronan. Biomacromolecules.

[50] Ouasti S., Donno R., Cellesi F., Sherratt M.J., Terenghi G., Tirelli N. (2011). Network connectivity, mechanical properties and cell adhesion for hyaluronic acid/PEG hydrogels. Biomaterials.

[51] Köwitsch A., Yang Y., Ma N., Kuntsche J., Mäder K., Groth T. (2011). Bioactivity of immobilized hyaluronic acid derivatives regarding protein adsorption and cell adhesion. Biotechnol. Appl. Biochem..

[52] Defife K.M., Hagen K.M., Clapper D.L., Anderson J.M. (1999). Photochemically immobilized polymer coatings: Effects on protein adsorption, cell adhesion, and leukocyte activation. J. Biomater. Sci. Polym. Ed..

[53] Chen C.-H., Cheng Y.-H., Chen S.-H., Chuang A.D.-C., Chen J.-P. (2021). Functional Hyaluronic Acid-Polylactic Acid/Silver Nanoparticles Core-Sheath Nanofiber Membranes for Prevention of Post-Operative Tendon Adhesion. Int. J. Mol. Sci..

[54] Hassanabad A.F., Zarzycki A.N., Jeon K., Deniset J.F., Fedak P.W.M. (2021). Post-Operative Adhesions: A Comprehensive Review of Mechanisms. Biomedicines.

[55] Seo Y., Kim J.W., Dho Y.S., Chowdhury T., Kim S., Park C.K. (2018). Evaluation of the safety and effectiveness of an alternative dural substitute using porcine pericardium for duraplasty in a large animal model. J. Clin. Neurosci..

[56] Dave J.M., Bayless K.J. (2014). Vimentin as an integral regulator of cell adhesion and endothelial sprouting. Microcirculation.

[57] Ivaska J., Pallari H.-M., Nevo J., Eriksson J.E. (2007). Novel functions of vimentin in cell adhesion, migration, and signaling. Exp. Cell Res..

[58] Mendez M.G., Kojima S., Goldman R.D. (2010). Vimentin induces changes in cell shape, motility, and adhesion during the epithelial to mesenchymal transition. FASEB J..

[59] Kim H., Nakamura F., Lee W., Shifrin Y., Arora P., McCulloch C.A. (2010). Filamin A is required for vimentin-mediated cell adhesion and spreading. Am. J. Physiol. Cell Physiol..

[60] Ostrowska-Podhorodecka Z., Ding I., Norouzi M., McCulloch C.A. (2022). Impact of Vimentin on Regulation of Cell Signaling and Matrix Remodeling. Front. Cell Dev. Biol..

[61] Cheng F., Shen Y., Mohanasundaram P., Lindström M., Ivaska J., Ny T., Eriksson J.E. (2016). Vimentin coordinates fibroblast proliferation and keratinocyte differentiation in wound healing via TGF-β-Slug signaling. Proc. Natl. Acad. Sci. USA.

[62] Pismensky S.V., Kalzhanov Z.R., Eliseeva M.Y., Kosmas I.P., Mynbaev O.A. (2011). Severe inflammatory reaction induced by peritoneal trauma is the key driving mechanism of postoperative adhesion formation. BMC Surg..

[63] Moreland L.W. (2003). Intra-articular hyaluronan (hyaluronic acid) and hylans for the treatment of osteoarthritis: Mechanisms of action. Arthritis Res. Ther..

[64] Mitsui Y., Gotoh M., Nakama K., Yamada T., Higuchi F., Nagata K. (2008). Hyaluronic acid inhibits mRNA expression of proinflammatory cytokines and cyclooxygenase-2/prostaglandin E2 production via CD44 in interleukin-1-stimulated subacromial synovial fibroblasts from patients with rotator cuff disease. J. Orthop. Res..

[65] Han G.-D., Kim J.-W., Noh S.-H., Kim S.-W., Jang E.-C., Nah J.-W., Lee Y.-G., Kim M.-K., Ito Y., Son T.-I. (2019). Potent anti-adhesion agent using a drug-eluting visible-light curable hyaluronic acid derivative. J. Ind. Eng. Chem..

