# Supplementary material for: Photo-Crosslinked Hyaluronic Acid/Carboxymethyl Cellulose Composite Hydrogel as a Dural Substitute to Prevent Post-Surgical Adhesion
Source: Int J Mol Sci. 2022 May 31;23(11):6177. doi: 10.3390/ijms23116177 (PMC9181059; doi:10.3390/ijms23116177)
Supplement: Supplementary file 1 [file ijms-23-06177-s001.zip › ijms-1734063-supplementary.pdf]

Supplementary Materials

Photo-Crosslinked Hyaluronic Acid/Carboxymethyl Cellulose Composite Hydrogel as a Dura Substitute to Prevent Post-Surgical Adhesion

Nuclear magnetic resonance (NMR) analysis

For NMR analysis, HAMA and CMCMA was dissolved in D_2O to obtain ^1H NMR spectra with Bruker Avance III HD 600 MHz NMR spectrometer (Billerica, MA, USA).

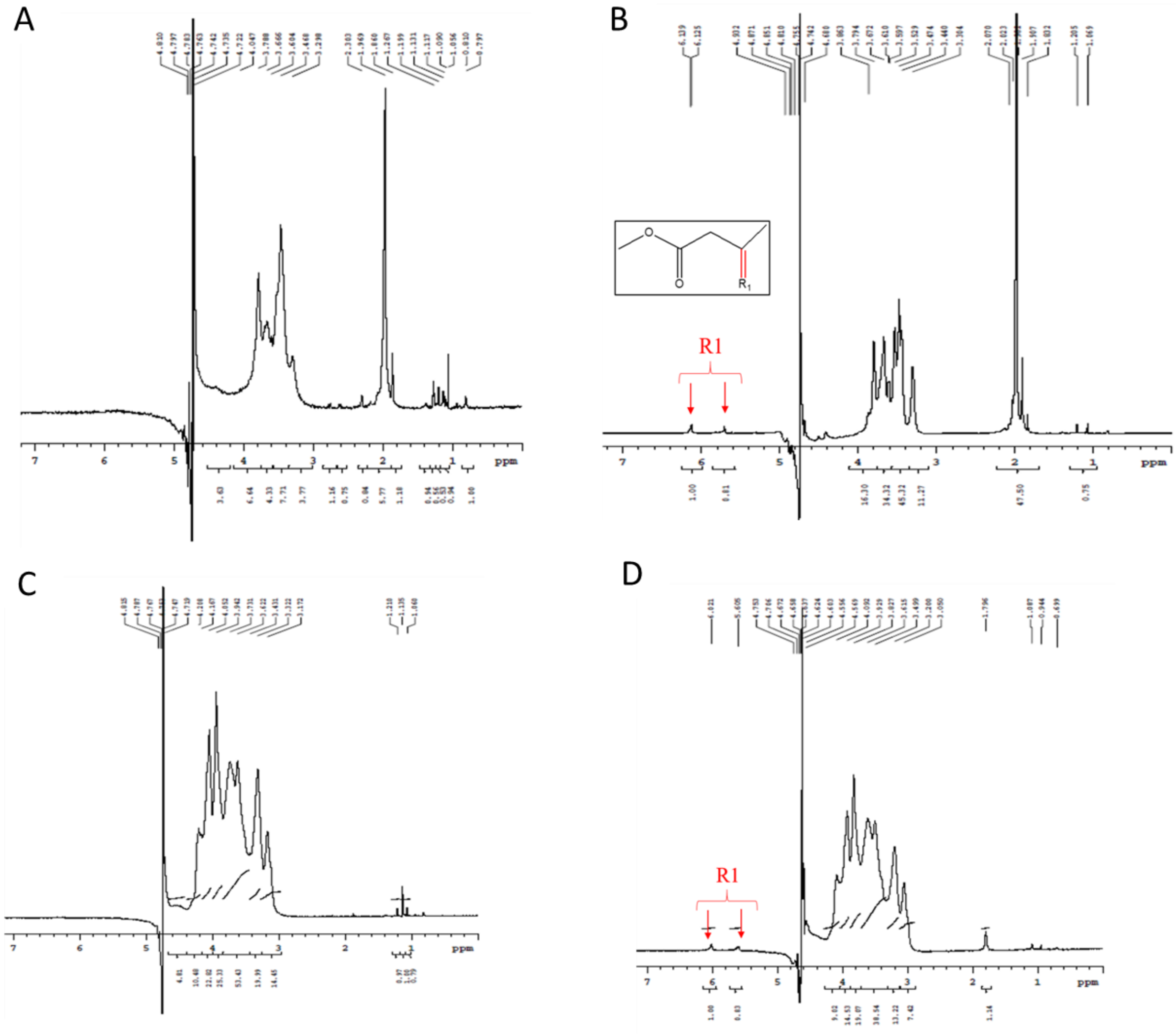

Figure S1. The ^1H NMR spectra of hyaluronic acid (HA) (A), hyaluronic acid methacrylate (HAMA) (B), carboxylmethyl cellulase (CMC) (C) and carboxylmethyl cellulase methacrylate (CMCMA) (D). R1 indicates methacrylate groups.
